# Comment to "Human cytomegalovirus seropositivity is associated with reduced patient survival during sepsis"

**DOI:** 10.1186/s13054-024-04914-2

**Published:** 2024-05-28

**Authors:** Zhihui Zhang, Xuesong Liu, Rong Zhang, Yimin Li, Xiaoqing Liu

**Affiliations:** 1 Department of Critical Care Medicine, State Key Laboratory of Respiratory Disease, National Clinical Research Center for Respiratory Disease, National Center for Respiratory Medicine, Guangzhou Institute of Respiratory Health, The First Affiliated Hospital of Guangzhou Medical University, Guangzhou, Guangdong People’s Republic of China; 2 Guangzhou Medical University, Guangzhou, Guangdong People’s Republic of China

We have read with great interest the clinical study and subsequent commentary by M Unterberg et al. published in Critical Care [1–3]. The study showed a robust correlation between human cytomegalovirus (HCMV) seropositivity and increased mortality. In addition, the study identified several biomarkers that could predict clinical outcomes in sepsis patients. This work marks a notable advancement in the field, shifting the focus from simply monitoring for HCMV reactivation to a more nuanced evaluation of HCMV serology as an indicator of latent infection. However, further refinement of certain details could have increased the study's depth.

First, a quantitative assessment of the prognostic impact of varying HCMV IgG levels on sepsis patient outcomes is warranted, rather than relying solely on qualitative serology. Active HCMV infection should be considered when IgG levels significantly exceed the reference value by more than fourfold [4, 5]. It is also important to recognize that the absence of HCMV IgG does not definitively rule out infection, particularly in critically ill patients with compromised immune function, such as those with septic shock, who may not be able to produce the necessary antibodies [6]. Thus, it is possible that the current study underestimated the incidence of HCMV seropositivity and reactivation. Furthermore, a comprehensive analysis of multiple biomarkers of HCMV infection, including IgG, IgM, DNAemia, and PP65 antigen, may provide a more detailed understanding of the status of infection.

Second, it is imperative to delineate the etiology and phase of sepsis. Differential analysis is necessary due to the differences in incidence and mortality rates between pulmonary and non-pulmonary sepsis. Our previous study found that patients with severe pneumonia made up nearly 70% of the immunocompetent patients with critical illness [7]. Furthermore, mortality risk associated with HCMV IgG detection may vary between the early stages (characterized by overwhelming inflammation) and the later stages(characterized by refractory inflammation, immunosuppression, and risk of secondary infections) [6]. Third, the study did not detail the antiviral prophylaxis or preemptive therapy regimens of the study population, which is a significant omission. The specific treatment protocols and medications used could significantly influence clinical outcomes [8–10]. Finally, proteomic sequencing methods might miss numerous biomarkers, however, their integration with transcriptomic or metabolomic sequencing may reveal the pathogenic mechanisms of HCMV in sepsis patients and provide novel therapeutic targets.

In conclusion, the study by Unterberg et al. attempts to predict the clinical prognosis of sepsis patients based on their HCMV serologic status, thus contributing positively to the advancement of the field. Nevertheless, more in-depth and meticulous research is essential to fully understand the interplay between HCMV serologic status and the clinical outcome of sepsis patients.

## Data Availability

Not applicable.
